# Supplementary material for: Rab11-dependent recycling of calcium channels is mediated by auxiliary subunit α2δ-1 but not α2δ-3
Source: Sci Rep. 2021 May 13;11:10256. doi: 10.1038/s41598-021-89820-1 (PMC8119971; doi:10.1038/s41598-021-89820-1)
Supplement: Supplementary file 1 — Supplementary Information. [file 41598_2021_89820_MOESM1_ESM.docx]

**Supplementary Figures**

**Fig. S1**. **Steady-state cell-surface Ca_V_2.2 is reduced by Rab11a(S25N) when expressed with α_2_δ-2**

**
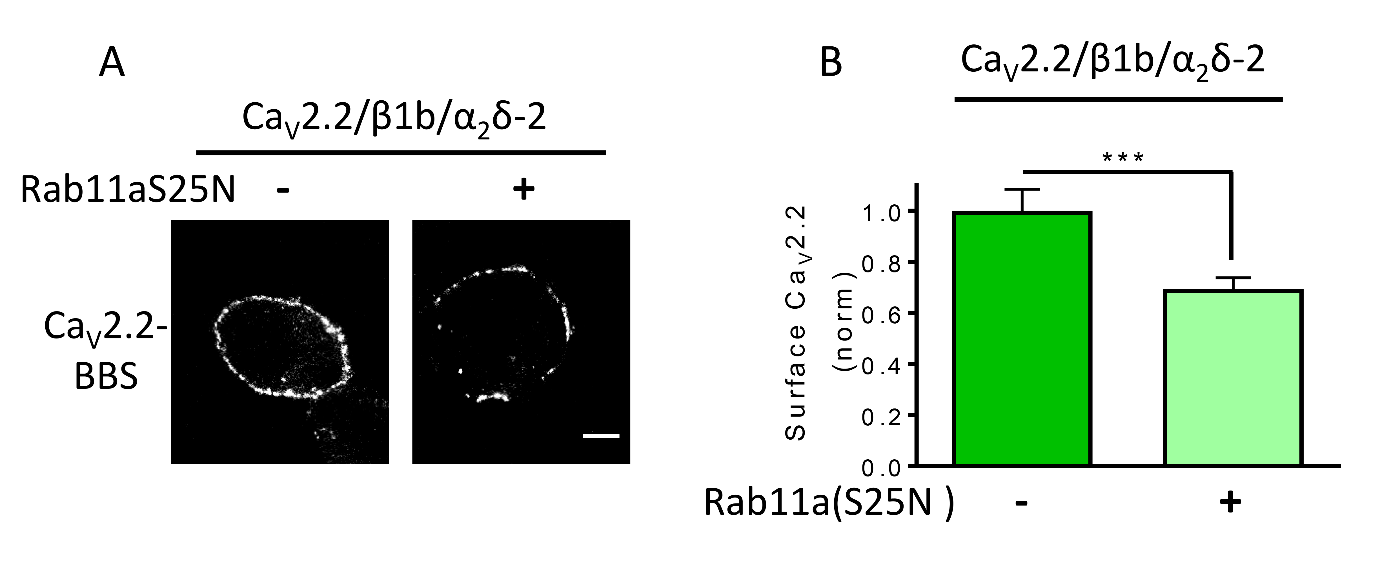
**

**(A)** Confocal images of cell-surface Ca_V_2.2-BBS/β1b expressed in N2a cells with α_2_δ-2 in the absence or presence of Rab11a(S25N). Scale bar = 5 µm.

**(B)** Normalized mean cell-surface Ca_V_2.2-BBS with β1b and either: α_2_δ-2 (green, n = 97 cells), α_2_δ-2 + Rab11a(S25N) (light green, n = 114 cells). Mean fluorescence intensity per cell was normalized to Ca_V_2.2/β1b/α_2_δ-2 controls, pooled from three separate transfections, and data are plotted as mean ± SEM values. Statistical significance was determined using Student’s unpaired t test, ****P* = 0.0009.

**Fig. S2 Rab11a(S25N) has no effect on steady-state inactivation of whole-cell Ca_V_2.2 currents when expressed with α_2_δ-1 or α_2_δ-3**


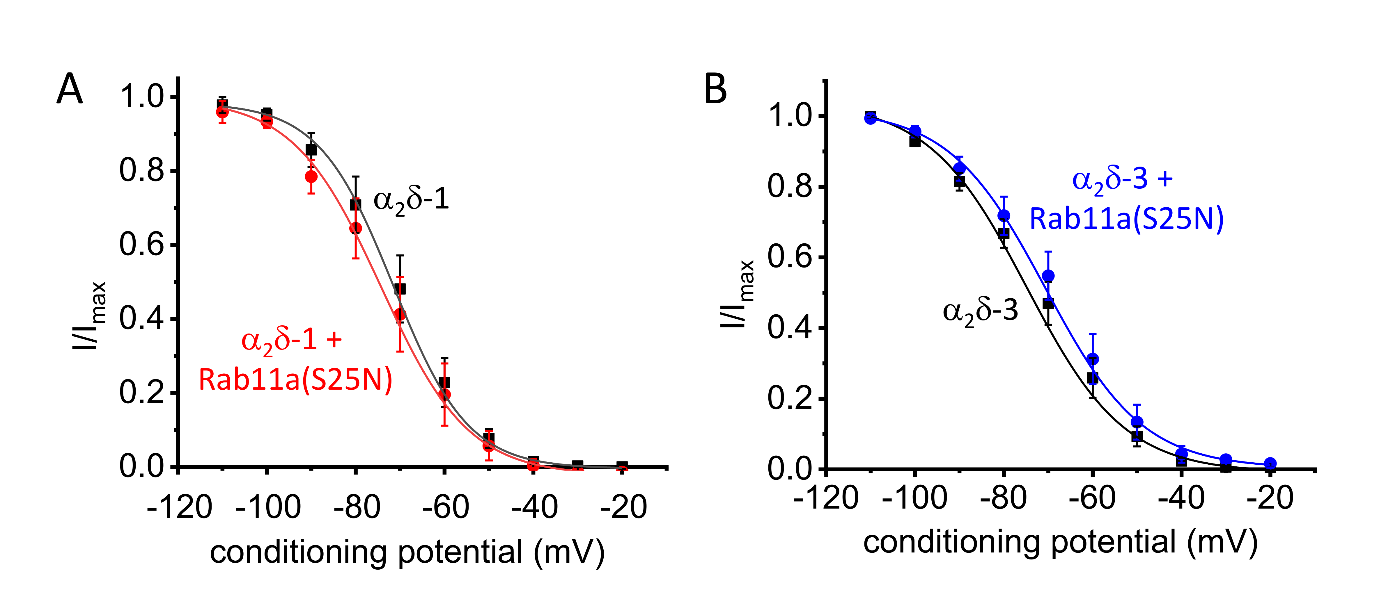


**(A)** Mean steady-state inactivation for Ca_V_2.2 with: β1b/α_2_δ-1 (black squares; n = 7 cells) or β1b/α_2_δ-1 + Rab11a(S25N) (red circles; n = 7 cells), fit to a Boltzmann function. The V_50, inact_ was -71.8 ± 3.6 mV and -73.8 ± 3.9 mV, respectively.

**(C)** Mean steady-state inactivation for Ca_V_2.2 with: β1b/α_2_δ-3 (black squares; n = 7 cells) or β1b/α_2_δ-3 + Rab11a(S25N) (blue circles; n = 7 cells), fit to a Boltzmann function, The V_50, inact_ was -71.5 ± 2.5 mV and -69.3 ± 3.4 mV, respectively.
